# Comparative Genomics of Novel *Agrobacterium* G3 Strains Isolated From the International Space Station and Description of *Agrobacterium tomkonis* sp. nov.

**DOI:** 10.3389/fmicb.2021.765943

**Published:** 2021-12-06

**Authors:** Nitin K. Singh, Céline Lavire, Joseph Nesme, Ludovic Vial, Xavier Nesme, Christopher E. Mason, Florent Lassalle, Kasthuri Venkateswaran

**Affiliations:** ^1^Biotechnology and Planetary Protection Group, Jet Propulsion Laboratory, California Institute of Technology, Pasadena, CA, United States; ^2^CNRS, INRAE, VetAgro Sup, UMR Ecologie Microbienne, Université de Lyon, Université Claude Bernard Lyon 1, Villeurbanne, France; ^3^Section of Microbiology, Department of Biology, University of Copenhagen, Copenhagen, Denmark; ^4^Department of Physiology and Biophysics, Weill Cornell Medicine, New York, NY, United States; ^5^Parasites and Microbes Programme, Wellcome Sanger Institute, Hinxton, United Kingdom

**Keywords:** *Agrobacterium tomkonis*, genomovar G3, ISS, metagenomics, phylogenomics, MLSA

## Abstract

Strains of *Agrobacterium* genomospecies 3 (i.e., genomovar G3 of the *Agrobacterium tumefaciens* species complex) have been previously isolated from diverse environments, including in association with plant roots, with algae, as part of a lignocellulose degrading community, from a hospital environment, as a human opportunistic pathogen, or as reported in this study, from a surface within the International Space Station. Polyphasic taxonomic methods revealed the relationship of *Agrobacterium* G3 strains to other *Agrobacterium* spp., which supports the description of a novel species. The G3 strains tested (*n* = 9) were phenotypically distinguishable among the strains from other genomospecies of the genus *Agrobacterium.* Phylogenetic analyses of the 16S rRNA gene, *gyr*B gene, multi-locus sequence analysis, and 1,089-gene core-genome gene concatenate concur that tested G3 strains belong to the *Agrobacterium* genus and they form a clade distinct from other validly described *Agrobacterium* species. The distinctiveness of this clade was confirmed by average nucleotide identity (ANI) and *in silico* digital DNA–DNA hybridization (dDDH) comparisons between the G3 tested strains and all known *Agrobacterium* species type strains, since obtained values were considerably below the 95% (ANI) and 70% (dDDH) thresholds used for the species delineation. According to the core-genome phylogeny and ANI comparisons, the closest relatives of G3 strains were *Agrobacterium* sp. strains UGM030330-04 and K599, members of a novel genomospecies we propose to call genomovar G21. Using this polyphasic approach, we characterized the phenotypic and genotypic synapomorphies of *Agrobacterium* G3, showing it is a *bona fide* bacterial species, well separated from previously named *Agrobacterium* species or other recognized genomic species. We thus propose the name *Agrobacterium tomkonis* for this species previously referred to as *Agrobacterium* genomospecies 3. The type strain of *A. tomkonis* is IIF1SW-B1^T^ (= LMG 32164 = NRRL B-65602). Comparative genomic analysis show *A. tomkonis* strains have species-specific genes associated with secretion of secondary metabolites, including an exopolysaccharide and putative adhesins and resistance to copper. *A. tomkonis* specific gene functions notably relate to surface adhesion and could be involved to colonize nutrient-poor and harsh habitats. The *A. tomkonis* strains from the ISS showed presence of a 40-kbp plasmid and several other potential mobile genetic elements detected that could also be part of conjugative elements or integrated prophages.

## Introduction

*Agrobacterium* species are firstly known for their pathogenic potential on plant, causing either crown gall or hairy root disease, depending on the presence of a tumor-inducing (Ti) or root-inducing (Ri) megaplasmid in their genome; however, most strains lack such plasmids and are not pathogenic. *Agrobacterium* species are common members of soil communities, and are efficient colonizers of the rhizospheres of a wide variety of plant hosts (Macrae et al., 1988; Matthysse and McMahan, 1998), with which they have a commensal relationship (Sanguin et al., 2006; Dessaux and Faure, 2018). *Agrobacterium* is a polyphyletic group, which taxonomy, originally aimed at reflecting the pathogenicity status of strains (Smith and Townsend, 1907), was revised multiple times, in the light of growing phenotypic and genotypic evidence, notably showing that the pathogenicity status was not correlated to the diversity of agrobacteria. Based on biochemical testing and DNA-DNA hybridization (DDH), *Agrobacterium* species were initially classified into three biovars (Keane et al., 1970; Kersters et al., 1973; Ophel and Kerr, 1987). *Agrobacterium* species belonging to biovar 1, as well as closely related species such as *Agrobacterium rubi* and *Agrobacterium larrymoorei* can be recognized by the presence of a linear chromid as the second major molecule of the genome, a synapomorphic trait enabled by the landmark acquisition of the *telA* gene (Ramírez-Bahena et al., 2014). The strains that belong to biovar 2 and biovar 3 are only distantly related to *Agrobacterium* biovar 1, which later led to their reclassification into the genera *Rhizobium* and *Allorhizobium*, respectively (Young et al., 2001; Mousavi et al., 2015). Further refinement of the taxonomy of *Agrobacterium* biovar 1, also known as the *Agrobacterium tumefaciens* species complex, was conducted based on whole-genome information using DDH, leading to the classification of strains into species-level units called genomic species or genomovars (Popoff et al., 1984). This classification framework was later validated and enriched by studies using amplified fragment-length polymorphism (AFLP), marker gene phylogeny, multi-locus sequence analysis (MLSA), comparative genome hybridization (CGH) and core-genome phylogeny (Portier et al., 2006; Costechareyre et al., 2010; Lassalle et al., 2011, 2017; Mousavi et al., 2015), as recently reviewed by Flores-Felix et al. (2020). At the time of writing this communication, the *Agrobacterium* genus contained 15 named species, of which 11 had validly published names. Moreover, nine out of 15 described genomic species within the *Agrobacterium* biovar 1 are not yet formally named: G3 (this study), G5, G6, G7 (“*Agrobacterium deltaense*”), G8 (“*Agrobacterium fabrum*”), G13, G15 (“*Agrobacterium viscosum”*), G19 and G20 (Mafakheri et al., 2019).

Several hundred microbial strains were isolated and identified in an ongoing investigation to map microbial diversity of the International Space Station (ISS; Checinska Sielaff et al., 2019). The ISS is a hermetically sealed closed system and their modules are at least 20-year-old, depending on their time of addition. The source of microorganisms is mainly through the human traffic, cargo transport and also associated with other experimental components. The air of the ISS is recirculated via an advanced environmental control system, and surfaces are maintained by implementing periodic cleaning. Previous attempts to isolate microorganisms from ISS environmental surfaces revealed that they generally consist of predominantly benign and commensal microorganisms (Mhatre et al., 2020; Bijlani et al., 2021), but potentially pathogenic microorganisms were also sporadically isolated (Knox et al., 2016; Singh et al., 2018a). In 2015, several bacterial strains were collected from the observation deck (Cupola) of the ISS, and whole-genome sequences (WGS) were generated for these isolates (Bharadwaj et al., 2020a,b; Bijlani et al., 2020a,b; Daudu et al., 2020a,b; Solomon et al., 2020; Simpson et al., 2021). Based on preliminary genomic analyses, three ISS strains were identified as belonging to the *Agrobacterium* genomospecies 3 (also known as *A. tumefaciens* genomovar G3 or *Agrobacterium* G3).

The first objective of this study is to describe the phylogenomic novelty and characterize the taxonomic affiliation of the three strains isolated from the ISS environment. We, therefore, assembled a dataset of complete genomes of *Agrobacterium* G3, including previously released sequences from five strains isolated from various geographical regions and environments: an eosin flask from a hospital environment in France (CFBP 6623, sequenced twice), a cave in Lechuguilla, United States (LC34), algae in the United Kingdom (SUL3), *Arabidopsis* roots in Germany (Root651), and bioprospecting for lignocellulolytic microbes and enzymes from natural, highly evolved plant biomass-degrading systems in the United States (UGM030330-04). We added two novel sequences from *Agrobacterium* G3 strains from our own collection, including an opportunistic pathogen isolated from cerebrospinal fluid (CFBP 6624) and one isolated from a tobacco plant rhizosphere (RTP8). In addition to traditional phenotype testing, molecular taxonomy utilizing 16S rRNA gene, *gyr*B, MLSA (*gyrB*, *parE*, *recA*, and *rpoB* genes), and core-gene-based phylogenic analyses were carried out to describe *Agrobacterium* G3 strains.

Second objective of this study is to perform a comparative genomic analysis of *Agrobacterium* G3 strains together with representatives of other *Agrobacterium* species (*n* = 41 genomes) to elucidate genomic complexity. Subsequently, experimental characterization of the plasmid content was carried out on the set of *Agrobacterium* strains available to us in culture, i.e., G3 strains IIF1SW-B1^T^, IIF1SW-B3, IIF1SW-B4, CFBP 6623, CFBP 6624, and RTP8 and reference strains “*A. fabrum* C58,” *Agrobacterium radiobacter* B6^T^, and *Agrobacterium pusense* CFBP 5494. Thirdly, a pangenome analysis was implemented to identify core homologous gene clusters specific to ISS isolates and other *Agrobacterium* G3 strains. Finally, the metagenome reads of ISS environmental surfaces (Singh et al., 2018b) were mined for the presence of *Agrobacterium* G3 species to understand the prevalence of these novel species in ISS.

## Results and Discussion

The three strains collected from ISS belonging to *Agrobacterium* G3 along with six other G3 strains were subjected to a polyphasic characterization to determine the variable, conserved or distinctive traits of this genomic species.

### Genome Characteristics of Novel Genome Sequences

Table 1 summarizes assembly statistics of all five novel ISS strains sequenced in this study. As an example, for strain IIF1SW-B1^T^, the Illumina NovaSeq platform yielded 1.8 × 10^7^ paired-end (PE) reads were reduced to 1.79 × 10^7^ PE reads after performing trimming and quality filtering. The assembled draft genome of ISS strains consisted of 76–81 contigs with a genome size of 6.25–6.29 × 10^6^ bp; the assembled genomes of CFBP 6624 and RTP8 had 35 and 38 contigs, for a size of 5.48 × 10^6^ bp and 5.44 × 10^6^ bp, respectively. The contig N50 size was 3.21–3.78 × 10^5^-bp, with a mean coverage of 100x for ISS strains, and 18x and 19x for strains CFBP 6624 and RTP8, respectively. G + C% of the five genomes was 59.12–59.21.

**TABLE 1:** Summary of the draft whole-genome sequences of several *Agrobacterium tomkonis* strains.

**Genome characteristics**	**IIF1SW-B1^T^**	**IIF1SW-B3**	**IIF1SW-B4**	**CFBP 6624**	**RTP8**
NCBI WGS accession	JABXYF000000000	JABXYG000000000	JABXYG000000000	JAFIRL000000000	JAFIRM000000000
# contigs (> = 0 bp)	137	154	150	44	55
# contigs (> = 1000 bp)	76	81	80	35	38
Total length (> = 1000 bp)	6,234,019	6,273,081	6,273,787	5,480,209	5,440,309
Largest contig	644,466	644,466	726,576	1,342,491	928,660
Total length	6,240,486	6,282,603	6,282,746	5,483,735	5,445,056
GC (%)	59.18	59.17	59.17	59.12	59.21
N50	350,004	350,004	354,854	378,069	320,860
# of genes	5,917	5,962	5,964	5,223	5,147
# CDS	5,863	5,908	5,910	5,175	5,098

### Phylogenetic Relationship of G3 Strains With Other *Agrobacterium* Species

**FIGURE 1:**
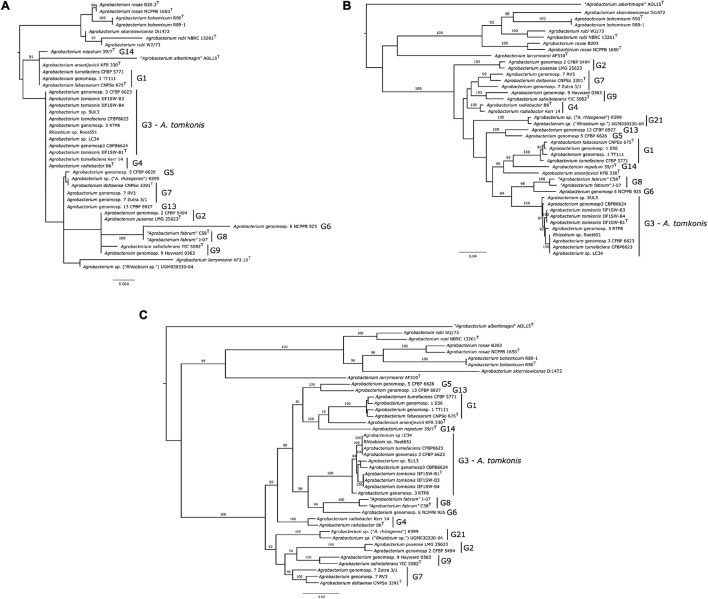
Phylogeny of 40 *Agrobacterium* spp. strains based on 16S rRNA gene, *gyrB*, and MLSA. Conserved marker gene phylogenies of 40 *Agrobacterium* distinct strains (41 when including the two published genome versions for strain CFBP 6623. **(A)** 16S rRNA gene phylogeny, based on 1,602 aligned positions), rooted with sequence from *Rhizobium leguminosarum* USDA 2370^T^; **(B)** *gyrB* gene phylogeny, based on 2,477 aligned positions; **(C)** multi-locus sequence analysis phylogeny, based on the concatenated alignments of genes *parE*, *gyrB*, *recA*, and *rpoB*, resulting into 9,968 aligned positions. All gene sequences were extracted from the 41 studied and then aligned with Clustal Omega (for coding genes, alignment was performed at the protein level and then reverse-translated into codons). Maximum-likelihood trees were inferred with RAxML-NG 1.0.0 under the model GTR + FO + G4m, taking the best of 20 independent inferences, started with 10 random trees and 10 parsimony-optimized tree. Branch supports were estimated with 200 Felsenstein bootstrap trees. Only branch support over 70% are displayed; full information on the trees are available at https://doi.org/10.6084/m9.figshare.14792148; https://doi.org/10.6084/m9.figshare.14792169; https://doi.org/10.6084/m9.figshare.14792100.

A Maximum Likelihood (ML) tree of 16S ribosomal RNA (rRNA) gene sequences shows that this marker is not able to resolve and distinguish all *Agrobacterium* species (Figure 1A). All strains from genomospecies G3 and G4; G7 and G13; as well as G2 and G9 were found to have identical 16S rRNA gene sequences. In addition, the support for the topology of the 16S tree is generally low, with only branches leading to species *A. rubi*, *Agrobacterium bohemicum* and the clade formed by *Agrobacterium* genomospecies G6 and G8 being well supported. In addition, the type strains of non-biovar 1 species *A. larrymoorei* and “*Agrobacterium albertimagni*” are positioned within the clade containing otherwise only *Agrobacterium* biovar 1 strains, indicating that these 16S alleles are likely the result of horizontal gene transfer events. Similar results showing high similarities or identical 16S rDNA sequences for *Agrobacterium rosae* NCPPB-1650^T^, *Agrobacterium skierniewicense* Di1472 (99.7%), and *A. rubi* NBRC 13261^T^ (99.5%) were reported (Kuzmanović et al., 2018). This altogether suggests that the 16S rRNA gene is not a good marker to identify *Agrobacterium* species or to study their relationships, as observed in other taxa (La Duc et al., 2004).

The gene *gyrB* provides a much better phylogenetic resolution of relationship among *Agrobacterium* species, with all except genomospecies G7 forming highly supported clades (Figure 1B). All nine strains belonging to G3 were grouped into one clade. The shallow grouping of species into clades is highly supported except G4, G7, and G9 strains, but deeper relationships within the *Agrobacterium* biovar 1 are not. This indicate that *gyrB* is a bona fide marker gene to identify and study the diversity of *Agrobacterium* species, even though its use as a marker for amplicon-based surveys has been shown to be impractical due to the presence of the paralogous gene *parE* in the genome rendering selective amplification difficult (Puławska and Kałużna, 2012).

The MLSA tree provides further resolution for *Agrobacterium* species, where strains belonging to various genomospecies were placed in a tight clade with high bootstrap values (Figure 1C). The relationships between species according to this MLSA tree are mostly in agreement with previous reports based on the concatenation of multiple loci or core-genome genes (Mousavi et al., 2014, 2015; Lassalle et al., 2017), indicating that this four-loci MLSA scheme (including *gyrB*, *parE*, *recA*, and *rpoB* genes) provides an efficient way to affiliate phylogenetic relationships among *Agrobacterium* species. All nine strains of *Agrobacterium* G3 formed a clade with a bootstrap value >88%.

**FIGURE 2:**
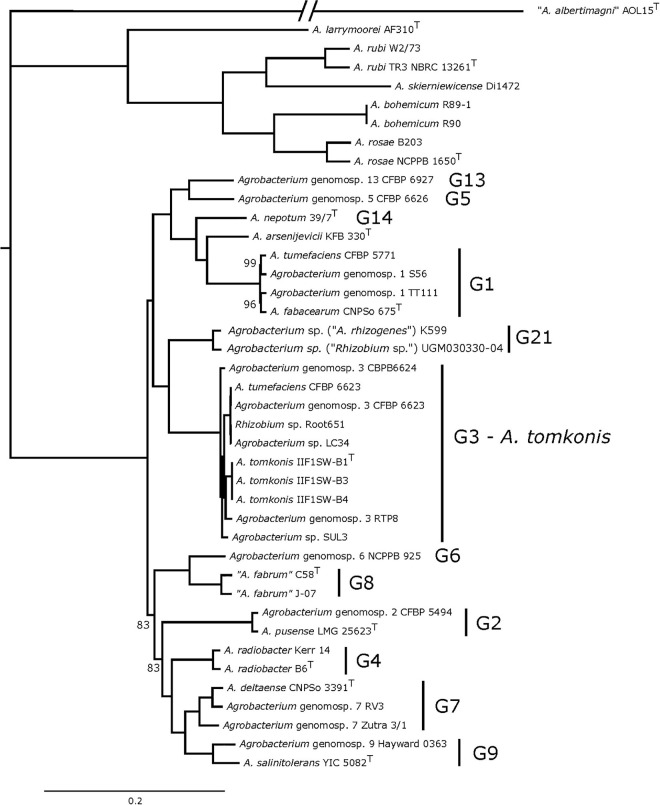
Phylogeny of 40 *Agrobacterium* spp. strains based on the concatenation of 1,089 core-genome genes. Maximum-likelihood phylogenetic tree of 40 *Agrobacterium* distinct strains (41 when including the two published genome versions for strain CFBP 6623) obtained using the bioinformatic pipeline Pantagruel (data available at https://doi.org/10.6084/m9.figshare.14792178). The tree was computed with RAxML 8.1.2 based on the concatenation of 1,089 core-genome gene alignments under the GTRCATX model, with 200 rapid bootstraps and rooted with RAxML tree-balance algorithm. All branch supports are 100%, unless displayed.

Finally, a tree based on the concatenation of 1,089 core-genome genes is fully resolved, with almost all branches having the highest support (Figure 2, bootstrap supports indicated if not 100%), and thus constitutes the gold standard for depicting the phylogenetic relationships of *Agrobacterium* species.

According to the *gyrB* and MLSA trees, the closest species to *Agrobacterium* G3 strains is the group formed of “*A. fabrum*” (G8) and *Agrobacterium* G6 strains, and these results were in accordance with previous reports (Lassalle et al., 2017). However, the core-genome tree revealed that the closest relatives were strains *Agrobacterium* sp. (*Rhizobium* sp.) UGM030330-04 and *Agrobacterium* sp. (*Agrobacterium rhizogenes*) K599 whereas strains belonging to genomovars G8 and G6 formed a different and distant clade (Figure 2). We note that strains UGM030330-04 and K599 are misnamed given these phylogenies clearly show that they are members of the *Agrobacterium* genus and thus not part of *Rhizobium*, nor of *A. rhizogenes*, which is a synonym of *R. rhizogenes*; they are thus more correctly referred to as *Agrobacterium* sp.

### Overall Genome Relatedness Indexes

All average nucleotide index (ANI) values among *Agrobacterium* G3 strains were over 97.5% whereas when comparing G3 strains to non-G3 strains, the ANI values were below 90.68% (Table 2 and Supplementary Table 1). The ANI values of *Agrobacterium* G3 strains and most closely related (as per core-genome) *Agrobacterium* sp. strains UGM030330-04 and K599 were ∼90.5% similarity. Similarly, the ANI values of *Agrobacterium* G3 strains with other closely related – according to *gyr*B and MLSA trees – *Agrobacterium* G6 (NCPPB 925) and G8 strains (J-07 and C58), exhibited ∼87.6% relatedness. Furthermore, average amino-acid identity (AAI) values among *Agrobacterium* G3 strains were over 97.6% while values between G3 and other agrobacteria were below 94%. Likewise, the digital DNA:DNA hybridization (dDDH) values among *Agrobacterium* G3 strains were over 83.6% while values between *Agrobacterium* G3 and other agrobacteria were 41.7% or below. More specifically the dDDH values were ∼41.7% for comparisons with strains UGM030330-04 and K599, 34.5% with G6 strains, and <33.9% with G8 strains. The ANI and dDDH values obtained for all the *Agrobacterium* G3 strains – including the ISS isolates – with other *Agrobacterium* species were below the threshold of 95% ANI (Yoon et al., 2017) and 70% dDDH values (Auch et al., 2010), which were established as standard for prokaryotic species delineation. We thus propose the name *Agrobacterium tomkonis* for the genomospecies 3 (= genomovar G3) of the *Agrobacterium* biovar 1 (i.e., *A. tumefaciens* species complex).

**TABLE 2:** List of *Agrobacterium* genomes used in this study and Average Nucleotide Identity (ANI), Average Amino acid Identity (AAI), and digital DNA-DNA Hybridization (dDDH) values of *A. tomkonis* IIF1SW-B1^T^ compared with all other tested *Agrobacterium* strains.

**Organism**	**GenBank #**	**OGRI values in comparison to *A. tomkonis* IIF1SW-B1^T^**			**Genomospecies**
		**ANI**	**AAI**	**dDDH**	
*Agrobacterium tomkonis* IIF1SW-B1^T^	JABXYF000000000	100.00	100.00	100.00	3
*Agrobacterium tomkonis* IIF1SW-B3	JABXYG000000000	100.00	100.00	100.00	3
*Agrobacterium tomkonis* IIF1SW-B4	JABXYH000000000	100.00	100.00	100.00	3
*Agrobacterium* genomospecies 3 CFBP 6623	GCF_900013535.1	98.08	97.62	84.90	3
*Agrobacterium* sp. (*Rhizobium* sp.) Root651	GCF_001427625.1	97.74	97.25	83.60	3
*Agrobacterium* genomospecies 3 LC34	GCF_001005815.1	97.82	97.77	83.50	3
*Agrobacterium* genomospecies 3 SUL3	GCF_001263295.1	97.64	97.73	83.90	3
*Agrobacterium* genomospecies 3 CFBP 6624	JAFIRL000000000	98.00	98.09	85.20	3
*Agrobacterium* genomospecies 3 RTP8	JAFIRM000000000	98.08	98.15	85.40	3
*Agrobacterium* genomospecies 3 CFBP 6623	GCA_005221385.1	98.12	97.61	84.80	3
*Agrobacterium* sp. (*Rhizobium* sp.) UGM030330-04	GCF_003208455.1	90.58	93.91	41.70	21
*Agrobacterium* sp. (*A. rhizogenes*) K599	GCF_002005205.3	90.51	93.39	41.50	21
*Agrobacterium* genomospecies 7 Zutra 3/1	GCF_900013515.1	88.63	91.71	36.70	7
*Agrobacterium* genomospecies 13 CFBP 6927		88.59	92.49	36.20	13
*Agrobacterium deltaense* CNPSo 3391	GCF_003931535.1	88.66	92.25	36.30	7
*Agrobacterium* genomospecies 7 RV3	GCF_900013505.1	88.56	91.77	36.30	7
*Agrobacterium* genomospecies 5 CFBP 6626	GCF_900012595.1	88.11	91.62	35.40	5
*Agrobacterium radiobacter* B6	GCF_900045375.1	87.97	90.95	35.40	4
*Agrobacterium radiobacter* Kerr 14	GCF_900011755.1	87.79	91.06	35.30	4
*Agrobacterium* genomospecies 6 NCPPB 925	GCF_900012625.1	87.54	90.09	34.50	6
*Agrobacterium fabrum* J-07	GCF_900013525.1	87.39	90.88	33.90	8
*Agrobacterium fabrum* C58	GCF_000092025.1	87.41	90.68	33.70	8
*Agrobacterium* genomospecies 9 Hayward 0363	GCF_900012565.1	87.76	91.98	34.20	9
*Agrobacterium salinitolerans* YIC 5082	GCF_002008225.1	87.55	91.18	33.90	9
*Agrobacterium arsenijevicii* KFB 330	GCF_000949895.1	87.04	89.42	33.70	^*^
*Agrobacterium nepotum* 39 7	GCF_000949865.1	87.21	89.99	33.60	14
*Agrobacterium tumefaciens* CFBP 5771	GCF_900039255.1	86.72	90.34	32.30	1
*Agrobacterium fabacearum* CNPSo 675	GCF_009649785.1	86.66	89.97	32.20	1
*Agrobacterium* genomospecies 1 TT111	GCF_900012575.1	86.60	89.84	32.30	1
*Agrobacterium* genomospecies 2 CFBP 5494	GCF_900013495.1	86.37	90.58	31.90	2
*Agrobacterium* genomospecies 1 S56	GCF_900014385.1	86.51	89.89	32.10	1
*Agrobacterium pusense* LMG 25623	GCF_900102105.1	86.51	90.59	31.80	2
*Agrobacterium larrymoorei* AF3 10 ATCC 51759	GCF_000518585.1	78.42	77.11	21.80	–
*Agrobacterium rubi* W2 73	GCF_001692345.1	77.93	75.37	21.30	–
*Agrobacterium rosae* B20 3	GCF_002915175.1	77.90	76.96	21.30	–
*Agrobacterium rubi* TR3 NBRC 13261	GCF_000739935.1	77.96	75.98	21.10	–
*Agrobacterium skierniewicense* Di1472	GCF_013320815.1	77.42	75.84	21.00	–
*Agrobacterium rosae* NCPPB 1650	GCF_002915195.1	77.81	76.35	21.20	–
*Agrobacterium bohemicum* R89-1	GCF_001562555.1	77.67	76.62	21.10	–
*Agrobacterium bohemicum* R90	GCF_002896715.1	77.51	76.47	21.00	–
*Agrobacterium albertimagni* AOL15	GCF_000300855.1	76.04	66.57	20.60	–

*A. arsenijevicii is part of the *A. tumefaciens* species complex but has not received a genomospecies number.

*Agrobacterium* sp. strains UGM030330-04 and K599 consistently form a distinct group in the *gyrB*, MLSA and core-genome tree; these two strains share an ANI of 98.11% and have <90.68% ANI with any other *Agrobacterium* strain. We therefore propose to group them into a new genomospecies of the *Agrobacterium* biovar 1, to be named genomovar G21, following the recently described genomovars G19 and G20 (Mafakheri et al., 2019).

### Phenotypic Characterization

Differential phenotypic characteristics of *A. tomkonis* strains with six other *Agrobacterium* species are given in Table 3 and Supplementary Table 2. The biochemical tests were highly variable among *Agrobacterium* species tested, in accordance with previous reports (Kuzmanović et al., 2018). However, we found a distinctive trait in that all *Agrobacterium* biovar 1 strains except those belonging to the *A. tomkonis* assimilated D-galacturonic acid. In addition, *A. tomkonis* strains tested assimilated the following carbon sources (Supplementary Table 2). D-maltose, D-trehalose, D-cellobiose, gentiobiose, sucrose, D-turanose, stachyose, D-raffinose, α-D-lactose, D-melibiose, β-methyl-D-glucoside, D-salicin, N-acetyl-D-glucosamine, N-acetyl-D-galactosamine, α-D-glucose, D-mannose, D-fructose, D-galactose, D-fucose, L-fucose, L-rhamnose, D-sorbitol, D-mannitol, D-arabitol, myo-inositol, glycerol, D-glucose- 6-PO4, D-fructose- 6-PO4, L-alanine, L-arginine, L-aspartic acid, L-glutamic acid, L-histidine, L-pyroglutamic acid, L-serine, pectin, D-gluconic acid, D-Glucuronic acid, quinic acid, methyl pyruvate, L-malic acid, γ-amino-butyric acid, propionic acid, acetic acid. Moreover, L-lactic acid, D-malic acid, bromosuccinic acid, β-hydroxy-D, L-butyric acid and acetoacetic acid support at least a weak metabolic activity. However, no metabolic activity was observed for *A. tomkonis* strains on N-acetyl-β-D-mannosamine, N-acetyl neuraminic acid, 3-Methyl Glucose, fusidic acid, D-serine, D-aspartic acid, D-galacturonic acid, L-galactonic acid lactone, mucic acid, D-saccharic acid, p-hydroxyphenylacetic acid, and sodium butyrate.

**TABLE 3:** Differential biochemical characteristics between *A. tomkonis* and closely related species.

**Biochemical tests**	***A. tomkonis***	***A. tumefaciens***	A. nepotum^*^	***“A. fabrum”***	***A. pusense***	***A. radiobacter***	A. larrymoorei^**^
	**IIF1SW-B1^T^**	**CFBP 5771**	**39/7^T^**	**C58**	**CFBP 5494**	**B6**	**AF3.10^T^**
D-Raffinose	+	+	+	+	+	+	−
α-D-Lactose	+	+	+	+	+	+	−
Acetic Acid	+	+	+	+	+	+	−
L-Arginine	+	+	−	−	−	+	nd
Methyl Pyruvate	+	w	+	−	w	w	+
N-Acetyl-D-Galactosamine	+	+	−	w	w	+	−
D-Galacturonic Acid	−	+	+	+	+	+	−
D-Saccharic Acid	−	−	−	−	−	−	+
D-Glucuronic Acid	w	w	+	+	+	+	−
L-Lactic Acid	w	+	−	+	+	+	+
D-Malic Acid	w	+	−	w	w	+	nd
Bromosuccinic Acid	w	w	−	−	−	−	+
Acetoacetic Acid	w	w	+	−	−	−	nd
Pectin	w	+	−	w	−	−	nd

*Data from Puławska et al. (2012); **Data from Bouzar and Jones (2001), Panday et al. (2011). +, positive; −, negative; w, weak reactions; nd, not determined.

Among various compounds tested, ISS *A. tomkonis* strains did not metabolize citric acid and Niaproof 4 (an anionic surfactant) whereas other *A. tomkonis* strains utilized them. Most of the compounds tested were metabolized unvaryingly by all ISS strains whereas strain IIF1SW-B3 was able to utilize lithium chloride as sole carbon source as that of other *A. tomkonis* strains tested. Similarly, strain IIF1SW-B3 was metabolizing Tween 40 (polysorbate, surfactant) but other *A. tomkonis* strains did not assimilate it. However, all six tested *A. tomkonis* strains are tolerant to troleandomycin, rifamycin SV, lincomycin, guanidine HCl, vancomycin, potassium tellurite, aztreonam, 1% sodium lactate, tetrazolium violet, and tetrazolium blue, but not to minocycline. Moreover, they all grew at pH 6 but not or only weakly at pH 5. All six tested *A. tomkonis* strains did not grow at 4% NaCl and beyond. The strains IIF1SW-B1^T^, IIF1SW-B4, and RTP8 tested on tomato produced no tumor (results not shown) and are therefore considered not pathogenic on tomato plant.

### Comparative Genomic Analysis and *A. tomkonis*-Specific Gene Content

Forty-nine genes were specifically found in *A. tomkonis* genomes. These genes were mostly interspersed in the genome and coded a range of functions (Supplementary Table 3), but a fraction clustered into three genomic islands, two on the linear chromosome and one on the largest of the two megaplasmids present in strain CFBP 6623 (pCFBP6623a). The first cluster of seven *A. tomkonis* specific genes clearly encodes the biosynthesis of a surface polysaccharide; the second encodes mostly proteins of unknown function, with one potentially related to type 6 secretion systems; the last *A. tomkonis* specific gene cluster on the plasmid encodes resistance to copper. Other *A. tomkonis* specific genes are scattered in the genome, but have related functions. There are notably two genes encoding ankyrin-repeat domain-containing proteins, as well as a protein containing an extracellular HAF repeat domain (InterPro domain IPR014262) and an autotransporter domain, all three exported to the cell surface and likely mediating attachment to an extracellular substrate. Together with the biosynthesis of a likely extracellular polysaccharide, these species-specific genes present a convergent function related to surface attachment and potentially resistance to stress from the external medium. This enrichment in the specific core-genome of *A. tomkonis* suggests natural selection was at work and that surface attachment may have a strong role in *A. tomkonis* ecology. Indeed, the first reported *A. tomkonis* strains CFBP 6623 and CFBP 6624 were isolated from within an eosin flask and from a human clinical sample (likely as an opportunist taking advantage of another pathogen infection), respectively (Popoff et al., 1984), showing already unusual colonization abilities for agrobacteria. Moreover, the strains recovered from the ISS were isolated from the surface of the observation dome (Cupola), which panel is made of aluminum with a polyurethane topcoat (e.g., Aeroglaze A276 or BMS10-60), an ultra-clean and smooth surface that carries very little microbial life. The independent isolation of G3 strains in such locations suggest that attachment to inhospitable surfaces may be a way for *A. tomkonis* to colonize new habitats where it can evade competition with other microbes.

**FIGURE 3:**
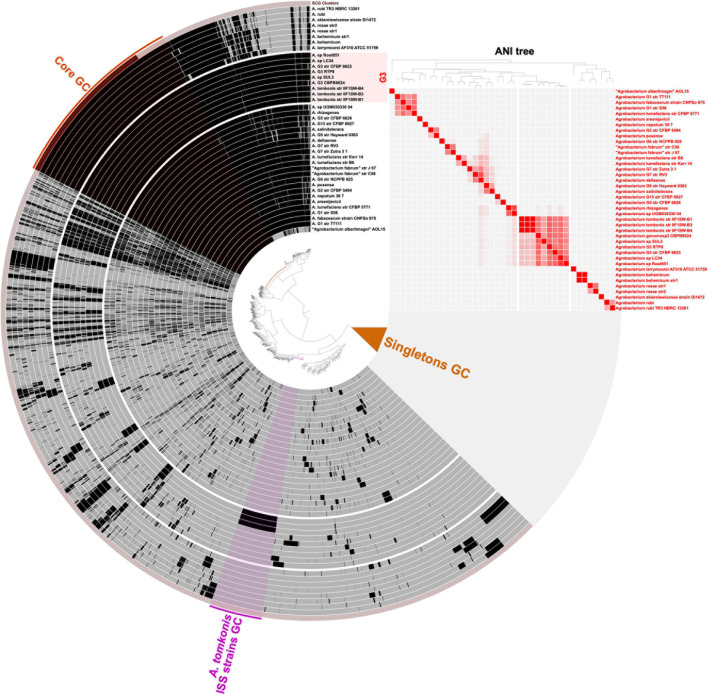
Visualization of the *Agrobacterium* pangenome. Genomes are ordered as layers using a tree based on average nucleotide identity (ANI) values matrix (Euclidean distance, Ward clustering). Color hue of ANI values are ranging from 70% ANI (white) to 100% ANI (red) and displayed as a heatmap on the right-side panel. Gene clusters are represented as vertical black bars indicating their presence in each genome layer. The central tree is ordering gene clusters based on their presence/absence in genomes using Euclidean distance and Ward clustering. The “Core GC” highlight indicates the gene clusters identified in all genomes. Gene clusters that are found in a single genome are collapsed as “Singletons GC.” The “*A. tomkonis* ISS strains GC” highlight indicates gene clusters found exclusively on the three strains isolated from the International Space Station. The figure was created in Anvi’o v6.

Similarly, the gene clustering analysis between the genomes of 40 different *Agrobacterium* strains allowed the identification of ISS strains-specific gene clusters (Figure 3 and Supplementary Table 4). We found 556 genes specifically present in the three ISS strains; we identified these genes based on their presence in all ISS strains and none of the 17 genomes in the background clade comprising the other *A. tomkonis* strains, all strains of *Agrobacterium arsenijevicii*, *Agrobacterium nepotum*, *Agrobacterium* genomospecies G1, G5, G13, and G2 (Supplementary Data online)^1^. Using a more relaxed criterion allowing presence of the genes in a maximum of 2 out the 17 genomes in the background clade, to allow the detection of genes specifically gained by the ancestor of the ISS strains but that may have independently introduced in close relatives via horizontal gene transfer, we found 838 genes (Supplementary Table 4), most of them located in large contiguous regions.

Among this wider set of ISS clone-specific genes, some were found to cover 10 entire contigs of the genome assembly of strain IIF1SW-B1^T^ (Supplementary Table 5), totaling to 347 genes over 613 kb of ISS clone-specific contigs, which possibly represent (parts of) extrachromosomal elements. Indeed, four of these ISS clone-specific contigs carried genes with functions associated to type 4 secretion systems (T4SS), with two seemingly complete clusters, indicating they may constitute conjugative elements such as plasmids or integrative and conjugative elements (ICE). Most of the other ISS clone-specific contigs carried transposase or genetic mobility-associated genes, suggesting they also constitute mobile genetic elements (MGEs). In addition, large contiguous portions of other contigs that were specific to the ISS strains carried transposase genes – often located at the extremity of the ISS clone-specific region – suggesting they may represent inserted MGEs such as transposons or genomic islands (Supplementary Tables 4, 5). In particular, the ISS clone-specific region of contigs #27 of strain IIF1SW-B1^T^ genome assembly clearly corresponds to a prophage.

Beyond genetic mobility, the ISS clone-specific genes displayed a range of other functions, which were enriched for a range of functions, some forming coherent cellular processes (Supplementary Table 6). Among the top enriched functions, we found a multi-copper oxidase, accompanied by a chaperone for its maturation and transporters that might allow the assimilation of copper ions from the environment; these functions may form a pathway for biosynthesis of a redox enzyme that could be involved in a respiratory chain. Another set of coherent functions were found to be involved in scavenging and uptake of a ferric ion-siderophore complex, as well as a non-ribosomal peptide synthases-polyketide synthases (NRPS/PKS) biosynthetic cluster, which might be involved in the biosynthesis of the siderophore.

### Plasmid Profiling

Based on migration of genomic DNA on Eckhardt gels (Vaudequin-Dransart et al., 1995), all *A. tomkonis* strains were evidenced to carry plasmids, although differences in size and number were observed between strains (Supplementary Figure 1). Indeed, strain CFBP 6624 presented a single plasmid of approximately 500 kb, whereas CFBP 6623, RTP8 and the three ISS strains showed one to three plasmids, with a common largest band around 260 kb. RTP8 and the three ISS strains have in common a 130-kb plasmid, and a 40-kb plasmid is present exclusively in the three ISS strains. The ISS strains do not harbor a pTi plasmid, as no classical *vir* genes were detected in their genomes (data not shown). Accordingly, no symptoms of crown-gall diseases were observed on injured tomato plants inoculated with any of the ISS strains (data not shown).

### *Agrobacterium tomkonis* Reads in International Space Station Metagenomes

In order to evaluate the relative abundance of *A. tomkonis* onboard the ISS, we performed metagenomic read recruitment obtained from ISS environmental DNA shotgun datasets (Singh et al., 2018b) with strain IIF1SW-B1^T^ genome as a reference. Visual inspection of the mapped read profiles showed that most recruited reads were located on conserved bacterial genes encoding products such as ribosomal RNA operons, ribosomal proteins, DNA-directed RNA polymerase *rpoB* or elongation factor Tu. Considering the high degree of conservation of these genes across distant bacterial lineages and the non-uniform read coverage of G3 contigs we conclude that despite being isolated from the same environment, *A. tomkonis* is not present in any detectable abundance in the ISS metagenomes.

In summary, *A. tomkonis* genomes are diverse, and notably carry a variety of plasmids, even though none of the G3 strain in our dataset carried a tumorigenic plasmid. As previously shown for other *Agrobacterium* genomic species (Lassalle et al., 2017), *A. tomkonis* strain genomes share a number of species-specific genes. *A. tomkonis* specific gene functions notably relate to surface adhesion and could be involved in the ecological specificity of *A. tomkonis* suggested by their varied source of isolation (Popoff et al., 1984). Indeed, while most species of the *Agrobacterium* biovar 1 are usually isolated from soils, plant rhizospheres or as plant pathogens inducing tumors (Barton et al., 2018; Dodueva et al., 2020; Weisberg et al., 2020), *A. tomkonis* strains never have been isolated as a plant pathogen, nor a tumor-inducing plasmid was observed in their genome, which we confirmed by negative infection tests on tomato plants. Even though the isolation of *A. tomkonis* strains RTP8 from a tobacco plant rhizosphere and Root651 from *Arabidopsis* roots prove that *A. tomkonis* strains are able to survive in plant rhizospheres, *A. tomkonis* strains were otherwise all isolated from a variety of unhospitable environments, including from an antiseptic flask, as an opportunistic pathogen of the human central nervous system, from a cave wall, and lastly from the inert surface of the ISS cupola. The ability to colonize these substrates, unusual for most agrobacteria, resonate with the finding that *A. tomkonis* genomes specifically carry genes involved in mediating attachment to surfaces, including production of putative adhesins and biofilm. This suggests a particular ability of *A. tomkonis* to colonize a different kind of habitats, that are even poorer in nutrients and harsher than soil, thus possibly escaping competition with other agrobacteria that are better at growing in richer environments such as plant rhizospheres.

The genomes of *A. tomkonis* strains isolated from the ISS, appear to be highly similar, suggesting they are clonally related. As such, they carry further specific genomic traits, including the presence of a 40-kbp plasmid observed via electrophoresis, as well as several other potential MGEs detected in their specific genomic content, with several ISS clone-specific contigs carrying conjugation genes (cumulated length 346 kbp) and more contigs that could also be part of conjugative elements or other types of MGEs such as integrated prophages. These ISS clone-specific genes also include many potentially adaptive genes, including putative pathways for respiratory chain biosynthesis and iron scavenging, but it is unclear what would be the selective advantage those genes could bring to the ISS strains in the context they were isolated from.

## Conclusion

Using a polyphasic approach, we characterized the phenotypic and genotypic synapomorphies of *Agrobacterium* G3 (*Agrobacterium* genomospecies 3), showing it is a *bona fide* bacterial species, well separated from previously named *Agrobacterium* species or other recognized genomic species. Based on this evidence, we propose to name this species *Agrobacterium tomkonis* (formal description below). In addition, we performed an in-depth investigation of the pangenome, notably identifying species-specific genes, allowing us to relate the description of this new species to hypotheses on its ecology. Within this novel species, we further characterized the clonal group of strain isolated from the ISS, showing it possesses its own set of specific genes and MGEs that could carry adaptive traits linked to its survival in the ISS environment.

### Description of *Agrobacterium tomkonis* sp. nov.

*Agrobacterium tomkonis* (tom. ko’ni.s. N.L gen. n. *tomkonis* referring to David Tomko, a well-known NASA Space Biology scientist who advanced space research in the United States). Cells are Gram-stain-negative, non-spore-forming, aerobic rods, 0.5–0.6 μm in width and 0.8–1.6 μm in length. Colonies are translucent and cream to white, with a diameter of 2–3 mm on R2A medium after incubation for 3 days at 25°C. Growth occurs at 20°C through 35°C, with an optimum temperature of 25°C. *A. tomkonis* strains grew at pH 6 but not or only weakly at pH 5 with a pH optimum at 7.5. All tested *A. tomkonis* strains failed to grow at 4% NaCl and beyond.

*A. tomkonis* strains metabolize D-maltose, D-trehalose, D-cellobiose, gentiobiose, sucrose, D-turanose, stachyose, D-raffinose, α-D-lactose, D-melibiose, β-methyl-D-glucoside, D-salicin, N-acetyl-D-glucosamine, N-acetyl-D-galactosamine, α-D-glucose, D-mannose, D-fructose, D-galactose, D-fucose, L-fucose, L-rhamnose, D-sorbitol, D-mannitol, D-arabitol, myo-inositol, glycerol, D-glucose- 6-PO4, D-fructose- 6-PO4, L-alanine, L-arginine, L-aspartic acid, L-glutamic acid, L-histidine, L-pyroglutamic acid, L-serine, pectin, D-gluconic acid, D-Glucuronic acid, quinic acid, methyl pyruvate, L-malic acid, γ-amino-butyric acid, propionic acid, acetic acid. However, did not utilize N-acetyl-β-D-mannosamine, N-acetyl neuraminic acid, 3-Methyl Glucose, fusidic acid, D-serine, D-aspartic acid, D-galacturonic acid, L-galactonic acid lactone, mucic acid, D-saccharic acid, p-hydroxyphenylacetic acid, and sodium butyrate.

*A. tomkonis* strains tested are tolerant to troleandomycin, rifamycin SV, lincomycin, guanidine HCl, vancomycin, potassium tellurite, aztreonam, 1% sodium lactate, tetrazolium violet, and tetrazolium blue, but not to minocycline.

The delineation of *A. tomkonis* from other *Agrobacterium* species was based on overall genome relatedness indexes (all *A. tomkonis* share ANI > 98% and dDDH > 84%), as well as based on well-supported clades in *gyrB* gene, MLSA (with *gyrB*, *parE*, *recA*, and *rpoB* genes) or core-genome phylogenies. The type strain, IIF1SW-B1^T^ (= LMG 32164 = NRRL B-65602), was isolated from the ISS Port panel of the Cupola, which is the observation deck for the crew. The type strain DNA G + C content is 59.18 mol% and its genome sequence is available from GenBank under WGS accession JABXYF000000000.

## Materials and Methods

### Sample Collection and Isolation of Bacteria

The sampling of ISS surfaces performed for this study took place within the United States on-orbit segments. Samples collected during this study were: Node 3 (Locations #1, #2, and #3), Node 1 (Locations #4 and #5), Permanent Multipurpose Module (Location #6), U.S. Laboratory (Location #7), and Node 2 (Locations #8 and control). A detailed description of the various locations sampled was published elsewhere (Singh et al., 2018b). Sample collection from ISS environmental surfaces, processing, and cultivation of bacteria have been previously reported (Checinska Sielaff et al., 2019). Three strains isolated during the second flight from Location #1 (Cupola) surfaces, were later identified as belonging to the genus *Agrobacterium* via 16S rRNA gene sequencing of the cultivated strains; these were designated: IIF1SW-B1^T^, IIF1SW-B3, and IIF1SW-B4.

### Phenotypic Characterization

Strains IIF1SW-B1^T^, IIF1SW-B3, IIF1SW-B4, CFBP 6623, CFBP 6624, and RTP8 strains were subjected for the phenotypic characterization. All strains are grown on yeast extract-peptone-glucose (YPG) medium and collected from the surface of the agar plates (Scortichini, 2012) before inoculation and screened for various organic substrate utilization using Biolog GEN III system (Biolog, Inc., Hayward, CA, United States) following manufacturer’s recommendations. Concurrently, two replicates of Biolog tests were carried out, and Biolog plates were incubated at 28°C for 48 h. After incubation, the OmniLog^TM^ system was used to measure carbon substrate utilization.

### Molecular Characterization of International Space Station Strains

A loopful of purified microbial culture (only ISS strains) was subjected to DNA extraction with the UltraClean DNA kit (MO BIO, Carlsbad, CA, United States) or Maxwell Automated System (Promega, Madison, WI, United States) as per manufacturer instructions. The extracted DNA was eluted in 50-μL of molecular grade water and stored at −20°C until further analysis. The 16S rRNA gene was amplified using the forward primer, 27F (5′-AGA GTT TGA TCC TGG CTC AG-3′) and the reverse primer, 1492R (5′-GGT TAC CTT GTT ACG ACT T-3′) (Checinska Sielaff et al., 2019) and PCR was performed with the following conditions: denaturation at 95°C for 5 min, followed by 35 cycles consisting of denaturation at 95°C for 50 s, annealing at 55°C for 50 s, and extension at 72°C for 1.5 min, and finalized by extension at 72°C for 10 min. The amplified products were treated with Antarctic phosphatase and exonuclease (New England Biolabs, Ipswich, MA, United States) to remove 5′- and 3′- phosphates from unused dNTPs before sequencing. Sequencing was performed by Macrogen (Rockville, MD, United States) using 27F and 1492R primers for *Bacteria*. The resulting sequences were assembled using SeqMan Pro from the DNASTAR Lasergene package (DNASTAR Inc., Madison, WI, United States). Bacterial sequences were searched against the EzTaxon-e database (Kim et al., 2012) and identified based on the closest percentage similarity (>98.6%) to previously identified microbial-type strains.

### Analysis of Plasmid Content

Plasmid profiles were determined by a modified Eckhardt agarose gel electrophoresis technique as previously described (Vial et al., 2006). Isolates were grown overnight in YPG medium at 28°C until the optical density at 600 nm reached 0.4. Electrophoresis was carried out at 5 V for 30 min and 90 V for 5 h at 4°C on a 0.75% agarose gel containing 1% sodium dodecyl sulfate. Plasmids sizes were estimated by comparison with those of “*A. fabrum*” C58 and *Allorhizobium vitis* S4 (Wood et al., 2001; Slater et al., 2009).

### Analysis of International Space Station Strains Pathogenicity on Tomato Plant

To test if the ISS G3 strains were plant-pathogen strains, 3-week-old tomato plants, cultivated with a photoperiod of 16 h/8 h light/dark in a greenhouse were stem-injured and inoculated with 10 μL of bacterial overnight culture (10^8^ CFU/ml). The plants were then incubated for 21 days, and the presence or absence of crown gall symptoms was established by visual inspection. “*A. fabrum*” C58 strain was used as the positive control.

### Whole Genome Sequencing of New *Agrobacterium* Strains

The WGS sequencing of the three ISS G3 strains was carried out as per established procedures (Singh et al., 2018a). Shotgun libraries were prepared using the Illumina Nextera Flex protocol (Singh et al., 2018b) and sequenced using NovaSeq 6000 S4 flow cell 2 × 150 PE sequencing kit. Verification of the quality of the raw sequencing data was carried out using FastQC v0.11.7 using default parameters^2^. Quality control for adapter trimming and quality filtering were performed using fastp v0.20.0 (Chen et al., 2018), and then SPAdes v3.11.1 (Bankevich et al., 2012) was used to assemble all the cleaned sequences. Fastp quality control was based on the following three parameters: (i) correction of mismatches in overlapped regions of paired-end reads, (ii) trimming of autodetected adapter sequences, and (iii) quality trimming at the 59 and 39 ends. To determine the quality of the assembled sequences, the number of contigs, the N50 value, and the total length were calculated using QUAST v5.0.2 (Gurevich et al., 2013). Default parameters were used for all software. The WGS sequencing of strains RTP8 and CFBP 6624 was carried out by Beckman Coulter Genomics (Takeley, Essex, United Kingdom). SPAdes v3.8.1 (Bankevich et al., 2012) was used to assemble all the cleaned sequences. All five new genomes were annotated using Prokka v1.14.5 (Seemann, 2014) with the following options: “–force –addgenes –compliant –usegenus.” Protein function was annotated by performing a blastp similarity search against a reference proteome database made of 405 high-quality genome assemblies obtained from the NCBI RefSeq, selecting all genomes belonging to the *Rhizobiaceae* family available as on 14th August 2018 that had a contig N50 greater than 98 kbp (Supplementary Table 7).

### Overall Genome Relatedness Index Calculations

The ANI (Yoon et al., 2017) between each pair of the 40 *Agrobacterium* genomes (41 genomes including the two published versions of strain CFBP 6623 genome) was calculated using pyANI (Pritchard et al., 2016). The dDDH analysis was performed using the Genome-to-Genome Distance Calculator 2.0 (GGDC 2.0) (Auch et al., 2010).

### Pangenome Analysis

We applied two separate pangenome analysis pipelines to obtain robust estimates of the sets of genes present and absent in each genome and to compute core and clade-specific gene sets for the various taxonomic groups represented in our dataset, including *Agrobacterium* biovar 1, *A. tomkonis* strains including ISS isolates.

(1) On one hand, a pangenomic Snakemake workflow (Köster and Rahmann, 2012) was run in anvi’o v6.2 (Eren et al., 2015). Briefly, gene clusters (GC) were defined by clustering all-versus-all open-reading frames BLAST similarity scores using the Markov Clustering (MCL) algorithm (Enright et al., 2002) with an inflation score of 8. Open reading frames were identified with Prodigal 2.6.3 in single mode (Hyatt et al., 2010). Open reading frames were annotated using COG and KOfam databases (Eren et al., 2015; Delmont and Eren, 2018). Results were displayed using *anvi-display-pan* function (Delmont and Eren, 2018). Functional enrichment in the ISS and G3 strain sets was obtained using *anvi-compute-functional-enrichment* function, as described in Shaiber et al. (2020), based on the COG annotation of open reading frames. Only enrichment for which FDR adjusted *p*-value < 0.05 are reported in Supplementary Table 5.

(2) On the other hand, a phylogenomic database was built with the Pantagruel pipeline (Lassalle et al., 2019), using the ‘‘usingGeneRax’’ branch of the code^3^ version “df79fee577b72389926a0138353fa657c11f3368.” The following pipeline tasks were performed: init, 00-03 and 05, leading to the clustering of homologous protein families with MMseqs2 (Steinegger and Söding, 2017), the alignment of their reverse-translated coding sequences with Clustal Omega (Sievers et al., 2011) and Python scripts using Biopython (Weisberg et al., 2020), and the computation of a maximum-likelihood (ML) phylogenetic tree with RAxML 8.1.2 (Stamatakis et al., 2005) based on the concatenation of 1,089 core-genome gene alignments, as previously described (Lassalle et al., 2021) (Supplementary Data File doi: 10.6084/m9.figshare.14792178). In addition, clade-specific sets of homologous gene clusters were determined using the “get_clade_specific_genes.r” script from Pantagruel package using the homologous gene family presence/absence matrix as input.

### Metagenome Sequence Reads Recruitment to Strain IIF1SW-B1

Metagenomic datasets (*n* = 42) previously obtained from environmental swabbing of the ISS (Singh et al., 2018b) were downloaded from NCBI-SRA BioProject PRJNA438545, quality filtered and mapped using BWA-MEM (Li and Durbin, 2009) against the genome assembly of strain IIF1SW-B1^T^. These steps were performed using anvi’o v6.2 (Eren et al., 2015) using anvi’o metagenomic workflow in reference mode and default parameters. Visual inspection of read mapping alignment was carried out using Artemis BamView (Carver et al., 2012).

### Phylogenetic Analysis of Marker Genes

For the 16S rRNA gene phylogenetic analysis, we extracted the 16S rRNA sequences from the genomes and added the reference 16S gene sequence from *Rhizobium leguminosarum* type strain USDA 2370^T^ (GenBank accession U29386.1) and then aligned all sequences with Clustal Omega (Sievers et al., 2011) (Supplementary Data File doi: 10.6084/m9.figshare.14792148). For the *gyrB* single-gene phylogenetic analyses, as well as multi-locus sequence analysis (MLSA), we used the coding sequence (CDS) alignments for the marker genes *parE*, *gyrB*, *recA* and *rpoB* from the Pantagruel database described above (see Supplementary Data Files available at doi: 10.6084/m9.figshare.14792169 and doi: 10.6084/m9.figshare.14792100). For the MLSA, we concatenated the CDS alignments of *parE*, *gyrB*, *recA*, and *rpoB* into a multi-locus alignment, totaling 9,968 positions. ML Phylogenies were then inferred based on these alignments with RAxML-NG 1.0.0 (Stamatakis et al., 2005) with the options “–model GTR + G4 –all,” implying the use of the model GTR + FO + G4m, and that 20 independent inferences were run, starting with 10 random trees and 10 parsimony-optimized tree, and retaining the best ML tree from these 20 inferences. Branch supports were estimated with 200 Felsenstein bootstrap trees. The 16S tree was rooted using *R. leguminosarum* USDA 2370^T^ sequence, whereas the *gyrB* and MLSA trees were rooted using the sequence from “*A. albertimagni*” AOL15 – a distant relative incorrectly classified in the *Agrobacterium* genus for which a classification in the newly proposed genus “*Peteryoungia*” was proposed (Kuzmanović et al., 2021).

## Data Availability Statement

The draft genome sequences of three ISS strains including type strainIIF1SW-B1^T^, as well as strains RTP8 and CFBP 6624, were deposited in NCBI. The genome sequences of isolates *A. tomknonis* IIF1SW-B1^T^, RTP8, and CFBP 6624 have been deposited to GenBank under the BioSample accessions SAMN15333190, SAMN17917486, and SAMN17918142, respectively; the WGS project accession numbers are also given in Table 1. The genome versions described in this paper are the first versions. Analysis intermediary data and result tables are available under the FigShare project available at https://figshare.com/account/home#/projects/115866 with individual items available at the following DOIs: 10.6084/m9.figshare.14792100, 10.6084/m9.figshare.14792148, 10.6084/m9.figshare.14792169, 10.6084/m9.figshare.14792178, 10.6084/m9.figshare.14883546, 10.6084/m9.figshare.14883516, 10.6084/m9.figshare.14798289, and 10.6084/m9.figshare.16782964.

## Author Contributions

CL, LV, XN, KV, and NS conceived and designed the microbiological experiments. NS, CL, and LV performed the microbiological experiments. CL and LV carried out the phenotypic assays Biolog based biochemical characterization. CL performed the plant pathogenicity experiments. LV performed plasmid content analyses. XN managed the *Agrobacterium* strain collection and the non-ISS strain genome sequencing project. NS and KV managed the ISS strain collection and the ISS strain genome sequencing project. FL, JN, and NS analyzed the data. NS analyzed the *de novo* assemblies, including contig alignment, and annotation checks. FL performed phylogenetic and phylogenomic analyses. JN performed metagenomic analysis. FL and JN performed pangenome analyses. FL and KV wrote the manuscript. All authors read and approved the final manuscript.

## Conflict of Interest

The authors declare that the research was conducted in the absence of any commercial or financial relationships that could be construed as a potential conflict of interest.

## Publisher’s Note

All claims expressed in this article are solely those of the authors and do not necessarily represent those of their affiliated organizations, or those of the publisher, the editors and the reviewers. Any product that may be evaluated in this article, or claim that may be made by its manufacturer, is not guaranteed or endorsed by the publisher.

## Footnotes

1
https://10.6084/m9.figshare.16782964

2
http://www.bioinformatics.babraham.ac.uk/projects/fastqc/

3
https://github.com/flass/pantagruel/tree/usingGeneRax

## Supplementary Material

Supplementary Figure 1 Plasmid profiles of *A. tomkonis* strains. Strains IIF1SW-B1, IIF1SW-B3 and IIF1SW-B4 showed the same plasmidic profil. Plasmids sizes were estimated by comparison with those of *A. fabrum* C58 and *A. vitis* S4 (data not shown). LC indicates the linear chromosome.

Supplementary Table 1 ANI matrix of all tested *Agrobacterium* strains.

Supplementary Table 2 Differential biochemical characteristics among *A. tomkonis* strains.

Supplementary Table 3 COG function enriched in 9 *Agrobacterium tomkonis* (G3) strains, compared to the 31 other *Agrobacterium* genomes in the dataset.

Supplementary Table 4 List of *Agrobacterium* clade-specific homologous gene families. Clade-specific genes are listed per clade; with each list starting by a hashtag and the clade id: “# cladeXX”. *A. tumefaciens* species complex/*Agrobacterium* biovar 1 is clade1; *A. tomkonis* is clade20; the clonal group of ISS strains is clade27. Genomes are listed using short codes, with the correspondence to strain names provided at the top.

Supplementary Table 5 List of contigs specific to the clonal group of *A. tomkonis* strains isolated from the ISS and their characteristics. The count of genes with genetic mobility-related functions is based on the Prokka annotation of proteins.

Supplementary Table 6 COG function enriched in 3 *Agrobacterium tomkonis* ISS strains, compared to the 37 other *Agrobacterium* genomes in the dataset.

Supplementary Table 7 List of genome assemblies used as reference to annotate the new *Agrobacterium* genomes with Prokka.
