# Supplementary figures and images for: Comparative Genomics of Novel Agrobacterium G3 Strains Isolated From the International Space Station and Description of Agrobacterium tomkonis sp. nov
Source: Front Microbiol. 2021 Dec 6;12:765943. doi: 10.3389/fmicb.2021.765943 (PMC8685578; doi:10.3389/fmicb.2021.765943)

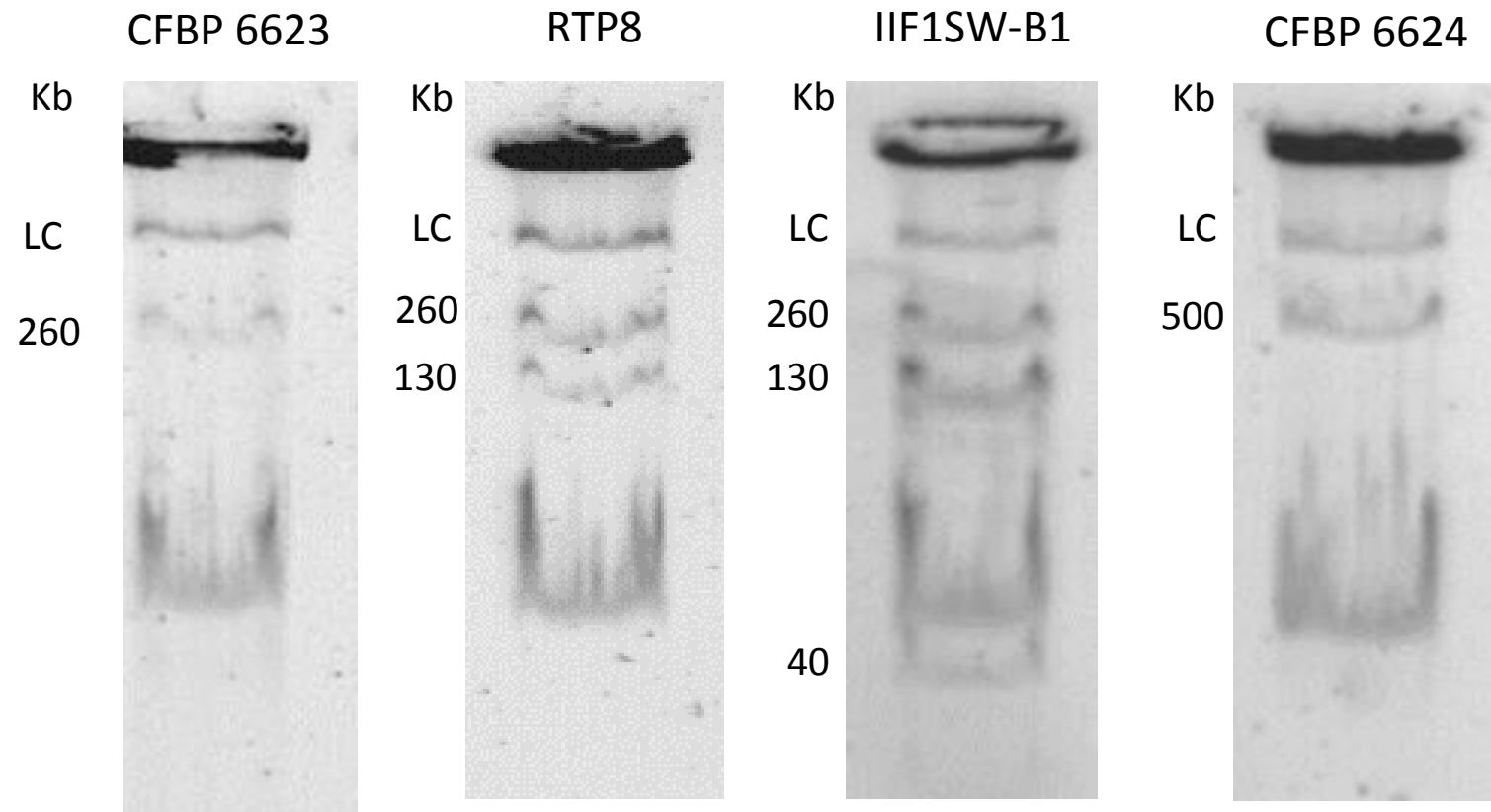

Supplement: Supplementary Figure 1 — Plasmid profiles of A. tomkonis strains. Strains IIF1SW-B1, IIF1SW-B3 and IIF1SW-B4 showed the same plasmidic profil. Plasmids sizes were estimated by comparison with those of A. fabrum C58 and A. vitis S4 (data not shown). LC indicates the linear chromosome. [file Image_1.pdf]
