# Supplementary material for: Comparative Genomics of Novel Agrobacterium G3 Strains Isolated From the International Space Station and Description of Agrobacterium tomkonis sp. nov
Source: Front Microbiol. 2021 Dec 6;12:765943. doi: 10.3389/fmicb.2021.765943 (PMC8685578; doi:10.3389/fmicb.2021.765943)
Supplement: Supplementary Table 2 — Differential biochemical characteristics among A. tomkonis strains. [file Data_Sheet_2.docx]

Supplemental Table S2. Differential biochemical characteristics among *A. tomkonis* strains.

|  | IIF1SW-B1^T^ | IIF1SW-B3 | IIF1SW-B4 | RTP8 | CFBP6623 | CFBP6624 |
| --- | --- | --- | --- | --- | --- | --- |
| Dextrin | +/w | +/w | +/w | +/w | - | - |
| D-Maltose | + | + | + | + | + | + |
| D-Trehalose | + | + | + | + | + | + |
| D-Cellobiose | + | + | + | + | + | + |
| Gentiobiose | + | + | + | + | + | + |
| Sucrose | + | + | + | + | + | + |
| D-Turanose | + | + | + | + | + | + |
| Stachyose | + | + | + | + | + | + |
| Positive Contr | + | + | + | + | + | + |
| pH 6 | + | + | + | + | + | + |
| pH 5 | - | - | +/w | +/w | - | - |
| D-Raffinose | + | + | + | + | + | + |
| α-D-Lactose | + | + | + | + | + | + |
| D-Melibiose | + | + | + | + | + | + |
| β-Methyl-D-Glucoside | + | + | + | + | + | + |
| D-Salicin | + | + | + | + | + | + |
| N-Acetyl-D-Glucosamin | + | + | + | + | + | + |
| N-Acetyl-β-D-Mannosamin | - | - | - | - | - | - |
| N-Acetyl-D-Galactosamin | + | + | + | + | + | + |
| N-Acetyl Neuraminic Ac | - | - | - | - | - | - |
| 1% NaCl | + | + | + | + | + | + |
| 4% NaCl | - | - | - | - | - | - |
| 8% NaCl | - | - | - | - | - | - |
| α-D-Glucose | + | + | + | + | + | + |
| D-Mannose | + | + | + | + | + | + |
| D-Fructose | + | + | + | + | + | + |
| D-Galactose | + | + | + | + | + | + |
| 3-Methyl Gluco | - | - | - | - | - | - |
| D-Fucose | + | + | + | + | + | + |
| L-Fucose | + | + | + | + | + | + |
| L-Rhamnos | + | + | + | + | + | + |
| Inosine | - | +/w | - | +/w | +/w | - |
| 1% Sodium Lactate | + | + | + | + | + | + |
| Fusidic Acid | - | - | - | - | - | - |
| D-Serine | - | - | - | - | - | - |
| D-Sorbitol | + | + | + | + | + | + |
| D-Mannitol | + | + | + | + | + | + |
| D-Arabitol | + | + | + | + | + | + |
| myo-Inositol | + | + | + | + | + | + |
| Glycerol | + | + | + | + | + | + |
| D-Glucose- 6-PO4 | + | + | + | + | + | + |
| D-Fructose- 6-PO4 | + | + | + | + | + | + |
| D-Aspartic Acid | - | - | - | - | - | - |
| D-Serine | - | - | - | - | - | - |
| Troleandomycin | + | + | + | + | + | + |
| Rifamycin SV | + | + | + | + | + | + |
| Minocycline | - | - | - | - | - | - |
| Gelatin | - | +/w | - | +/w | - | - |
| Glycyl-L-Proline | - | +/w | +/w | +/w | +/w | +/w |
| L-Alanine | + | + | + | + | + | + |
| L-Arginine | + | + | + | + | + | + |
| L-Aspartic Acid | + | + | + | + | + | + |
| L-Glutamic Acid | + | + | + | + | + | + |
| L-Histidine | + | + | + | + | + | + |
| L-Pyroglutamic Acid | + | + | + | + | + | + |
| L-Serine | + | + | + | + | + | + |
| Lincomycin | + | + | + | + | + | + |
| Guanidine HCl | + | + | + | + | + | + |
| Niaproof | - | - | - | + | + | + |
| Pectin | + | + | + | + | + | + |
| D-Galacturonic Acid | - | - | - | - | - | - |
| L-Galactonic Acid Lactone | - | - | - | - | - | - |
| D-Gluconic Acid | + | + | + | + | + | + |
| D-Glucuronic Acid | + | + | + | + | + | + |
| Glucuronamide | - | +/w | - | +/w | - | - |
| Mucic Acid | - | - | - | - | - | - |
| Quinic Acid | + | + | + | + | + | + |
| D-Saccharic Acid | - | - | - | - | - | - |
| Vancomycin | + | + | + | + | + | + |
| Tetrazolium Violet | + | + | + | + | + | + |
| Tetrazolium Blue | + | + | + | + | + | + |
| p-Hydroxy- Phenylacetic Acid | - | - | - | +/w | - | - |
| Methyl Pyruvate | + | + | + | + | + | + |
| D-Lactic Acid Methyl Ester | - | + | +/w | +/w | +/w | - |
| L-Lactic Acid | +/w | + | +/w | + | +/w | + |
| Citric Acid | - | - | - | +/w | + | + |
| α-Keto-Glutaric Acid | - | +/w | - | +/w | +/w | - |
| D-Malic Acid | +/w | + | +/w | + | + | +/w |
| L-Malic Aci | + | + | + | + | + | + |
| Bromo-Succinic Acid | +/w | + | + | +/w | +/w | +/w |
| Nalidixic Acicd | - | - | - | +/w | +/w | - |
| Lithium Chloride | - | + | - | + | + | +/w |
| Potassium Tellurite | + | + | + | + | + | + |
| Tween 40 | - | + | - | +/w | +/w | - |
| γ-Amino-Butryric Acid | + | + | + | + | + | +/w |
| α-Hydroxy-Butyric Acid | - | +/w | - | +/w | +/w | - |
| β-Hydroxy-D,L-Butyric Acid | + | + | + | + | + | +/w |
| α-Keto-Butyric Acid | - | +/w | - | +/w | - | - |
| Acetoacetic Acid | + | +/w | +/w | +/w | + | +/w |
| Propionic Acid | + | + | + | + | + | + |
| Acetic Acid | + | + | + | + | + | + |
| Formic Acid | - | +/w | +/w | +/w | - | - |
| Aztreonam | + | + | + | + | + | + |
| Sodium Butyrate | - | - | - | - | - | - |
| Sodium Bromate | - | +/w | - | +/w | +/w | - |
