# Supplementary material for: Comparative Genomics of Novel Agrobacterium G3 Strains Isolated From the International Space Station and Description of Agrobacterium tomkonis sp. nov
Source: Front Microbiol. 2021 Dec 6;12:765943. doi: 10.3389/fmicb.2021.765943 (PMC8685578; doi:10.3389/fmicb.2021.765943)
Supplement: Supplementary Table 7 — List of genome assemblies used as reference to annotate the new Agrobacterium genomes with Prokka. [file Data_Sheet_7.docx]

GCF_000009265.1_ASM926v1

GCF_000016265.1_ASM1626v1

GCF_000016285.1_ASM1628v1

GCF_000020265.1_ASM2026v1

GCF_000021345.1_ASM2134v1

GCF_000023185.1_ASM2318v1

GCF_000092025.1_ASM9202v1

GCF_000092045.1_ASM9204v1

GCF_000172695.2_ASM17269v2

GCF_000172715.2_ASM17271v2

GCF_000172795.2_ASM17279v2

GCF_000192635.1_ASM19263v1

GCF_000214615.1_Agro31749_1.0

GCF_000219665.1_ASM21966v2

GCF_000233975.1_ASM23397v2

GCF_000236125.1_ASM23612v2

GCF_000268285.2_RPHCH2410v2

GCF_000271785.1_ASM27178v1

GCF_000271805.1_ASM27180v1

GCF_000271825.1_ASM27182v1

GCF_000271845.1_ASM27184v1

GCF_000281735.1_ASM28173v1

GCF_000282035.1_CF122.fsa

GCF_000282095.2_Rhizobium.strCF080_v2.0

GCF_000292525.1_ASM29252v1

GCF_000298315.2_ASM29831v2

GCF_000300855.1_ASM30085v1

GCF_000312665.1_ASM31266v1

GCF_000330885.1_ASM33088v1

GCF_000349865.1_Cherry1.0

GCF_000359745.1_Rhizobium_sp.

GCF_000371905.1_ASM37190v1

GCF_000372105.1_ASM37210v1

GCF_000372205.1_ASM37220v1

GCF_000372305.1_ASM37230v1

GCF_000373025.1_ASM37302v1

GCF_000373285.1_ASM37328v1

GCF_000373325.1_ASM37332v1

GCF_000373425.1_ASM37342v1

GCF_000375705.1_ASM37570v1

GCF_000377185.1_ASM37718v1

GCF_000377565.1_ASM37756v1

GCF_000379005.1_ASM37900v1

GCF_000379605.1_ASM37960v1

GCF_000381165.1_ASM38116v1

GCF_000384555.1_ASM38455v1

GCF_000385155.1_ASM38515v1

GCF_000419705.1_ASM41970v1

GCF_000419745.1_ASM41974v1

GCF_000419765.1_ASM41976v1

GCF_000426285.1_ASM42628v1

GCF_000427705.1_ASM42770v1

GCF_000427765.1_ASM42776v1

GCF_000427925.1_ASM42792v1

GCF_000427945.1_ASM42794v1

GCF_000427985.1_ASM42798v1

GCF_000430465.1_ASM43046v1

GCF_000439225.1_AtWRT31V1.0

GCF_000442435.1_ASM44243v1

GCF_000442985.1_AtP4V1.0

GCF_000482285.1_ASM48228v1

GCF_000499645.1_IRBG74

GCF_000510625.1_ASM51062v1

GCF_000515375.1_ASM51537v1

GCF_000517045.1_ASM51704v1

GCF_000517605.1_ASM51760v1

GCF_000518585.1_ASM51858v1

GCF_000518785.1_ASM51878v1

GCF_000520875.1_ASM52087v1

GCF_000576515.1_ASM57651v1

GCF_000577275.1_LPU83

GCF_000621665.1_ASM62166v1

GCF_000696095.1_ASM69609v1

GCF_000698845.1_ASM69884v1

GCF_000702405.1_ASM70240v1

GCF_000705135.2_Draft_genome_assembly_of_Agrobacterium_rhizogenes_ATCC15834

GCF_000705355.1_SOAPdenovo_for_version_1.05_of_Rhizobium_marinus_MGL06

GCF_000705615.1_CCGM1.1

GCF_000722615.1_ASM72261v1

GCF_000722625.1_ASM72262v1

GCF_000723345.1_AGROS2_V1

GCF_000730975.1_ASM73097v1

GCF_000731295.1_RG1141_Ch_pA_pB

GCF_000731315.1_RG540_Ch_pA

GCF_000732195.1_ASM73219v1

GCF_000739935.1_ASM73993v1

GCF_000744505.1_ASM74450v1

GCF_000744565.1_ASM74456v1

GCF_000744575.1_ASM74457v1

GCF_000745255.1_ASM74525v1

GCF_000745575.1_ASM74557v1

GCF_000745655.1_ASM74565v1

GCF_000745725.1_ASM74572v1

GCF_000745735.1_ASM74573v1

GCF_000745785.1_ASM74578v1

GCF_000746265.1_ASM74626v1

GCF_000746325.1_ASM74632v1

GCF_000746335.1_ASM74633v1

GCF_000746385.1_ASM74638v1

GCF_000757525.1_RRTA

GCF_000758445.1_ASM75844v1

GCF_000769405.1_ASM76940v1

GCF_000799685.1_ASM79968v1

GCF_000799715.1_ASM79971v1

GCF_000799755.1_ASM79975v1

GCF_000799775.1_ASM79977v1

GCF_000799845.1_ASM79984v1

GCF_000799895.1_ASM79989v1

GCF_000799905.1_ASM79990v1

GCF_000799925.1_ASM79992v1

GCF_000799945.1_ASM79994v1

GCF_000799975.1_ASM79997v1

GCF_000799985.1_ASM79998v1

GCF_000800035.1_ASM80003v1

GCF_000800135.1_ASM80013v1

GCF_000816125.1_ASM81612v1

GCF_000816845.1_ASM81684v1

GCF_000834635.1_ASM83463v1

GCF_000931365.1_ASM93136v1

GCF_000949865.1_ASM94986v1

GCF_000949895.1_ASM94989v1

GCF_000959075.1_ASM95907v1

GCF_000966435.1_ASM96643v1

GCF_000967425.1_ASM96742v1

GCF_000971565.1_ASM97156v1

GCF_000985875.1_NgalHAMBI1146Draft

GCF_000985895.1_NgalHAMBI2610Draft

GCF_000985915.1_NgalHAMBI1145Draft

GCF_000985955.1_NgalHAMBI2566Draft

GCF_000985975.1_NgalHAMBI1189Draft

GCF_000985995.1_NgalHAMBI2605Draft

GCF_000986015.1_NgalHAMBI490Draft

GCF_000986035.1_NgalHAMBI2427Draft

GCF_001005815.1_ASM100581v1

GCF_001005825.1_ASM100582v1

GCF_001187535.1_ASM118753v1

GCF_001263295.1_SUL3v1

GCF_001295785.1_ASM129578v1

GCF_001296045.1_ASM129604v1

GCF_001297245.1_ASM129724v1

GCF_001421235.1_Leaf202

GCF_001421985.1_Leaf68

GCF_001422245.1_Leaf262

GCF_001423215.1_Leaf321

GCF_001423425.1_Leaf306

GCF_001423445.1_Leaf311

GCF_001424045.1_Leaf371

GCF_001424065.1_Leaf384

GCF_001424085.1_Leaf386

GCF_001424245.1_Leaf155

GCF_001424325.1_Leaf341

GCF_001424505.1_Leaf391

GCF_001424945.1_Root1203

GCF_001424965.1_Root1204

GCF_001424985.1_Root1212

GCF_001425145.1_Root1240

GCF_001425345.1_Leaf383

GCF_001425605.1_Leaf453

GCF_001426265.1_Root1220

GCF_001426565.1_Root482

GCF_001426665.1_Root1334

GCF_001426685.1_Root483D2

GCF_001427205.1_Root491

GCF_001427345.1_Root149

GCF_001427405.1_Root564

GCF_001427625.1_Root651

GCF_001428925.1_Root268

GCF_001429075.1_Root73

GCF_001429245.1_Root274

GCF_001429725.1_Root708

GCF_001526505.1_ASM152650v1

GCF_001526525.1_ASM152652v1

GCF_001526545.1_ASM152654v1

GCF_001526565.1_ASM152656v1

GCF_001526585.1_ASM152658v1

GCF_001541305.1_ASM154130v1

GCF_001541315.1_ASM154131v1

GCF_001541345.2_ASM154134v2

GCF_001542405.1_ASM154240v1

GCF_001551895.1_ASM155189v1

GCF_001562555.1_ASM156255v1

GCF_001612535.1_ASM161253v1

GCF_001632995.1_ASM163299v1

GCF_001641425.1_ASM164142v1

GCF_001644925.1_GHKF11

GCF_001649535.1_ASM164953v1

GCF_001652265.1_ASM165226v1

GCF_001657485.1_ASM165748v1

GCF_001662055.1_ASM166205v1

GCF_001662075.1_ASM166207v1

GCF_001662105.1_ASM166210v1

GCF_001662125.1_ASM166212v1

GCF_001664085.1_ASM166408v1

GCF_001664105.1_ASM166410v1

GCF_001664125.1_ASM166412v1

GCF_001664145.1_ASM166414v1

GCF_001664165.1_ASM166416v1

GCF_001664185.1_ASM166418v1

GCF_001664205.1_ASM166420v1

GCF_001664225.1_ASM166422v1

GCF_001664245.1_ASM166424v1

GCF_001664265.1_ASM166426v1

GCF_001664285.1_ASM166428v1

GCF_001664305.1_ASM166430v1

GCF_001664325.1_ASM166432v1

GCF_001664345.1_ASM166434v1

GCF_001664365.1_ASM166436v1

GCF_001664385.1_ASM166438v1

GCF_001664405.1_ASM166440v1

GCF_001664425.1_ASM166442v1

GCF_001664445.1_ASM166444v1

GCF_001664465.1_ASM166446v1

GCF_001664485.1_ASM166448v1

GCF_001664525.1_ASM166452v1

GCF_001664545.1_ASM166454v1

GCF_001675075.1_ASM167507v1

GCF_001675095.1_ASM167509v1

GCF_001675175.1_ASM167517v1

GCF_001679565.1_ASM167956v1

GCF_001687365.1_ASM168736v1

GCF_001691455.1_ASM169145v1

GCF_001692155.1_ASM169215v1

GCF_001692185.1_ASM169218v1

GCF_001692195.1_ASM169219v1

GCF_001692215.1_ASM169221v1

GCF_001692245.1_ASM169224v1

GCF_001692265.1_ASM169226v1

GCF_001692275.1_ASM169227v1

GCF_001692285.1_ASM169228v1

GCF_001692325.1_ASM169232v1

GCF_001692345.1_ASM169234v1

GCF_001692365.1_ASM169236v1

GCF_001692405.1_ASM169240v1

GCF_001692425.1_ASM169242v1

GCF_001713475.1_ASM171347v1

GCF_001729315.1_ASM172931v1

GCF_001758275.1_ASM175827v1

GCF_001758305.1_ASM175830v1

GCF_001854685.1_ASM185468v1

GCF_001854865.1_ASM185486v1

GCF_001860085.1_ASM186008v1

GCF_001860105.1_ASM186010v1

GCF_001860125.1_ASM186012v1

GCF_001860135.1_ASM186013v1

GCF_001860165.1_ASM186016v1

GCF_001860185.1_ASM186018v1

GCF_001860205.1_ASM186020v1

GCF_001860215.1_ASM186021v1

GCF_001885585.1_ASM188558v1

GCF_001890425.1_ASM189042v1

GCF_001908375.1_ASM190837v1

GCF_001908615.1_ASM190861v1

GCF_001927265.1_ASM192726v1

GCF_001931685.1_ASM193168v1

GCF_001938935.1_ASM193893v1

GCF_001938945.1_ASM193894v1

GCF_001938985.1_ASM193898v1

GCF_001939045.1_ASM193904v1

GCF_001975795.1_ASM197579v1

GCF_001999485.1_ASM199948v1

GCF_002000045.1_ASM200004v1

GCF_002005205.2_ASM200520v2

GCF_002008155.1_ASM200815v1

GCF_002008165.1_ASM200816v1

GCF_002008205.1_ASM200820v1

GCF_002008215.1_ASM200821v1

GCF_002008225.1_ASM200822v1

GCF_002008275.1_ASM200827v1

GCF_002008365.1_ASM200836v1

GCF_002078095.1_ASM207809v1

GCF_002117485.1_ASM211748v1

GCF_002117725.1_ASM211772v1

GCF_002119845.1_ASM211984v1

GCF_002179795.1_ASM217979v1

GCF_002179845.1_ASM217984v1

GCF_002204185.1_ASM220418v1

GCF_002211285.1_ASM221128v1

GCF_002211295.1_ASM221129v1

GCF_002211305.1_ASM221130v1

GCF_002211315.1_ASM221131v1

GCF_002211365.1_ASM221136v1

GCF_002211385.1_ASM221138v1

GCF_002211395.1_ASM221139v1

GCF_002240185.1_ASM224018v1

GCF_002243365.1_ASM224336v1

GCF_002245285.1_ASM224528v1

GCF_002277895.1_ASM227789v1

GCF_002355115.1_ASM235511v1

GCF_002406495.1_L101

GCF_002406505.1_JJW1

GCF_002531595.1_ASM253159v1

GCF_002531615.1_ASM253161v1

GCF_002531635.1_ASM253163v1

GCF_002531645.1_ASM253164v1

GCF_002531655.1_ASM253165v1

GCF_002531695.1_ASM253169v1

GCF_002531715.1_ASM253171v1

GCF_002531725.1_ASM253172v1

GCF_002531755.1_ASM253175v1

GCF_002531775.1_ASM253177v1

GCF_002531795.1_ASM253179v1

GCF_002531805.1_ASM253180v1

GCF_002531825.1_ASM253182v1

GCF_002531855.1_ASM253185v1

GCF_002531885.1_ASM253188v1

GCF_002531905.1_ASM253190v1

GCF_002531935.1_ASM253193v1

GCF_002531955.1_ASM253195v1

GCF_002591665.1_ASM259166v1

GCF_002600635.1_ASM260063v1

GCF_002750855.1_ASM275085v1

GCF_002812325.1_ASM281232v1

GCF_002814035.1_ASM281403v1

GCF_002855515.1_ASM285551v1

GCF_002894505.1_ASM289450v1

GCF_002896715.1_ASM289671v1

GCF_002914525.1_ASM291452v1

GCF_002915175.1_ASM291517v1

GCF_002915195.1_ASM291519v1

GCF_002933635.1_ASM293363v1

GCF_002943835.1_ASM294383v1

GCF_002944315.1_ASM294431v1

GCF_002948295.1_ASM294829v1

GCF_002953715.1_ASM295371v1

GCF_002968535.1_ASM296853v1

GCF_002968555.1_ASM296855v1

GCF_002968565.1_ASM296856v1

GCF_002968575.1_ASM296857v1

GCF_002968635.1_ASM296863v1

GCF_002968675.1_ASM296867v1

GCF_002968795.1_ASM296879v1

GCF_002968815.1_ASM296881v1

GCF_002968845.1_ASM296884v1

GCF_003001755.1_ASM300175v1

GCF_003013335.1_ASM301333v1

GCF_003024605.1_ASM302460v1

GCF_003025035.1_ASM302503v1

GCF_003031125.1_ASM303112v1

GCF_003031555.1_ASM303155v1

GCF_003031575.1_ASM303157v1

GCF_003053405.1_ASM305340v1

GCF_003058385.1_ASM305838v1

GCF_003122325.1_ASM312232v1

GCF_003150695.1_ASM315069v1

GCF_003205195.1_ASM320519v1

GCF_003208455.1_ASM320845v1

GCF_003217095.1_ASM321709v1

GCF_003217115.1_ASM321711v1

GCF_003217135.1_ASM321713v1

GCF_003217305.1_ASM321730v1

GCF_003240565.1_ASM324056v1

GCF_003240585.1_ASM324058v1

GCF_003259755.1_ASM325975v1

GCF_003261015.1_ASM326101v1

GCF_003269135.1_ASM326913v1

GCF_003287875.1_ASM328787v1

GCF_003290405.1_ASM329040v1

GCF_003350285.1_ASM335028v1

GCF_003351165.1_ASM335116v1

GCF_003351175.1_ASM335117v1

GCF_003351185.1_ASM335118v1

GCF_900011755.1_AHY_PRJEB12188_v1

GCF_900012565.1_AHY_PRJEB12195_v1

GCF_900012575.1_AHY_PRJEB12182_v1

GCF_900012585.1_AHY_PRJEB12191_v1

GCF_900012595.1_AHY_PRJEB12189_v1

GCF_900012605.1_AHY_PRJEB12186_v1

GCF_900012615.1_AHY_PRJEB12196_v1

GCF_900012625.1_AHY_PRJEB12190_v1

GCF_900013495.1_AHY_PRJEB12183_v1

GCF_900013505.1_AHY_PRJEB12192_v1

GCF_900013515.1_AHY_PRJEB12193_v1

GCF_900013525.1_AHY_PRJEB12194_v1

GCF_900013535.1_AHY_PRJEB12185_v1

GCF_900014385.1_AHY_PRJEB12181_v1

GCF_900039255.1_AHY_PRJEB12180_v1

GCF_900045375.1_AHY_PRJEB12187_v1

GCF_900067135.1_IMG-taxon_2602042090_annotated_assembly

GCF_900094545.1_IMG-taxon_2617270742_annotated_assembly

GCF_900094555.1_IMG-taxon_2615840609_annotated_assembly

GCF_900094565.1_IMG-taxon_2615840626_annotated_assembly

GCF_900094585.1_IMG-taxon_2615840698_annotated_assembly

GCF_900094625.1_IMG-taxon_2615840624_annotated_assembly

GCF_900099775.1_IMG-taxon_2596583570_annotated_assembly

GCF_900100235.1_IMG-taxon_2690315628_annotated_assembly

GCF_900100645.1_Agro2

GCF_900100825.1_Agro1

GCF_900102105.1_IMG-taxon_2671180227_annotated_assembly

GCF_900104545.1_IMG-taxon_2600255308_annotated_assembly

GCF_900108425.1_Tibet

GCF_900108545.1_IMG-taxon_2600254933_annotated_assembly

GCF_900109605.1_IMG-taxon_2617270885_annotated_assembly

GCF_900110205.1_IMG-taxon_2671180023_annotated_assembly

GCF_900111085.1_IMG-taxon_2600255279_annotated_assembly

GCF_900111275.1_IMG-taxon_2599185156_annotated_assembly

GCF_900111905.1_IMG-taxon_2599185236_annotated_assembly

GCF_900156055.1_IMG-taxon_2708742397_annotated_assembly

GCF_900169785.1_ASM90016978v1

GCF_900176345.1_IMG-taxon_2716884867_annotated_assembly

GCF_900177415.1_IMG-taxon_2588254269_annotated_assembly

GCF_900188475.1_IMG-taxon_2521172559_annotated_assembly

GCF_900215255.1_IMG-taxon_2617270923_annotated_assembly

GCF_900215635.1_IMG-taxon_2619618868_annotated_assembly

GCF_900220975.1_IMG-taxon_2740891857_annotated_assembly

GCF_900492185.1_T2.30D-1.1
